# Can we determine anterior hip coverage from pelvic anteroposterior radiographs? A study of patients with hip dysplasia

**DOI:** 10.1186/s12891-023-06624-2

**Published:** 2023-06-24

**Authors:** Hui Cheng, Zhendong Zhang, Wei Sun, Ningtao Ren, Dianzhong Luo, Yong Li, Jianli Zhang, Hong Zhang

**Affiliations:** Senior Department of Orthopaedics, the, Fourth Medical Center , PLA General Hospital, Beijing, 100048 China

**Keywords:** Hip dysplasia, Anterior coverage, Periacetabular osteotomy, Pelvic anteroposterior radiographs, False profile radiograph

## Abstract

**Purpose:**

Insufficient coverage causes hip joint instability and results in hip pain. Anterior hip coverage can be determined on both pelvic anteroposterior (AP) radiographs and false profile (FP) radiographs. Four parameters are commonly used to determine the anterior coverage on pelvic AP radiographs: the crossover index, crossover sign, anterior wall index (AWI), and rule of thirds. This study aims to clarify the relationship between these 4 parameters on AP radiographs and the anterior center edge angle (ACEA) on FP radiographs.

**Methods:**

In this study, 53 patients who underwent periacetabular osteotomy for hip dysplasia at our center between July 2020 and October 2020 were retrospectively reviewed. Four parameters on AP radiographs and the ACEA on FP radiographs before surgery and 6 months after surgery were measured and compared for each hip.

**Results:**

Upon examining the 53 hips in this study, there was no correlation between either the crossover index and the ACEA (P = 0.66) or the crossover sign before surgery. The postoperative correlation between the crossover index and the ACEA was weak (r = 0.36, P = 0.007), and that between the crossover sign and the ACEA was moderate (r = 0.41, P = 0.003). There was a weak correlation between the AWI and ACEA both before (r = 0.288, P = 0.036) and after (r = 0.349, P = 0.011) the operation. Evaluation of the anterior coverage by the rule of thirds was also not consistent when determining the anterior coverage with the ACEA.

**Conclusion:**

Anterior coverage on AP radiographs is largely inconsistent with ACEA on FP radiographs, especially before the surgery. It is recommended to take FP radiographs routinely for determining anterior hip coverage.

## Introduction

Hip dysplasia is a hip deformity that is characterized by insufficient acetabular coverage of the femoral head, which causes hip joint instability and results in hip pain [1]. However, the lack of anterior hip coverage can cause symptoms around the hip joint as well as lack of lateral coverage [2–4]. Following the increasing understanding of the 3D morphology of the hip joint, an increasing emphasis has been placed on the importance of anterior hip coverage by not only joint surgeons [2, 5, 6] but also pediatric orthopedic surgeons [3, 7] and sports medicine surgeons [8–10], especially for borderline hip dysplasia [3, 9, 11–13].

Accurate measuring of anterior hip coverage is crucial for determining if hip preservation surgery is necessary, as well as for estimating the outcome of such surgery [3]. Although 3D measurements are the most accurate method for determining the 3D morphology of the acetabulum, pelvic tilt caused by the supine position in CT scanning reduces its ability to reproduce the weightbearing state of the hips [14–21], influencing the measurements of anterior coverage [16, 18, 22–24]. Furthermore, few surgeons can measure 3D acetabular coverage directly on an imaging system in the hospital, which is even more impossible during surgery, so 2D measurements of anterior coverage are still widely used in clinical practice.

Radiographs taken in the standing position reproduce the weight-bearing status of a hip joint. A standing false profile (FP) radiograph is one of the most intuitive methods to see the supporting part of the acetabulum and to measure the anterior coverage of the hip joint. It is widely accepted by hip surgeons that the anterior center edged angle (ACEA) measured on FP radiographs can be utilized in determining the anterior coverage of the hip joint, although there is one study that found that the ACEA (measured by a different method) may have limited ability to predict three-dimensional coverage of the femoral head in patients with developmental dysplasia of the hip [25]. An association between ACEA and clinical outcomes has been revealed in previous studies [3, 9, 12, 26–29], with it being a powerful predictor of a surgery [3].

Methods of estimating anterior hip coverage by identifying the anterior acetabular rim on a standing pelvic AP graph are also used in clinical practice, particularly when standard FP radiographs are not available, such as during the surgery. Currently, the parameters that are commonly used to determine anterior coverage on pelvic AP radiographs include the crossover index, crossover sign [30], anterior wall index (AWI) [31], and rule of thirds [32].

However, the correlation between the parameters and ACEA is far from strong [30, 33, 34]. TA previous study has shown that different parameters of anterior coverage present different abilities to predict clinical results [3].

Therefore, this study aims to define the correlation between the crossover index and the ACEA, the correlation between the crossover sign and the ACEA, the correlation between the anterior wall index (AWI) and the ACEA, and the correlation between the rule of thirds and the ACEA.

### Patients and methods

This retrospective study reviewed patients who underwent isolated periacetabular osteotomy (PAO) for hip dysplasia our center between July and October of 2020. The Institutional Review Board of the hospital approved the study. Patients were included if they met specific criteria, including closed triangular cartilage of the acetabulum, simple dysplasia or mild hip subluxation (Crowe type 0–1) [35], Tönnis osteoarthritis grade 0–1 [36], and good to excellent congruence of the hip joint [37]. Additionally, they must have had at least 6 months of follow-up. Exclusion criteria included insufficient or poor-quality imaging data, a history of surgery on the affected hip joint, severe hip joint deformity caused by neurological or muscular diseases (such as poliomyelitis or cerebral palsy), and an irregular shape of the femoral head preventing accurate measurement of imaging data (such as coxa plana or multiple epiphyseal dysplasia).

Pelvic standing AP radiographs and standard FP radiographs [25] were taken for each patient before surgery and at 6 months after the surgery when the patients could walk with full body weight. Because standing radiography can recreate the state of the hip during body weight loading, the pelvic tilt was not normalized [38].

**Fig. 1:**
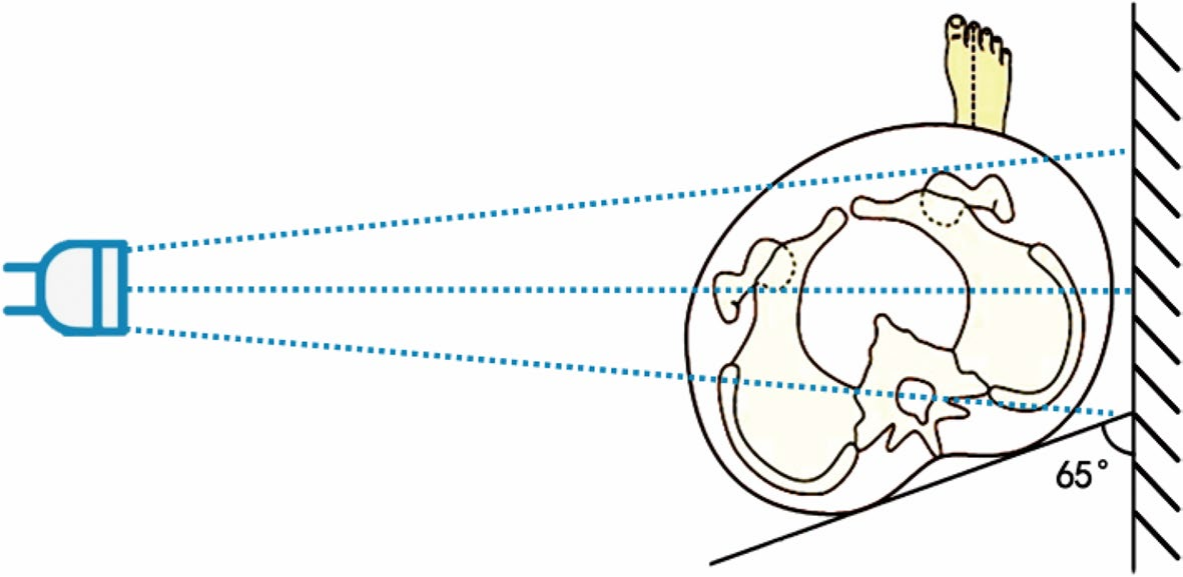
The false profile radiographs were taken according to the introduction by Professor Lequesne. The patient was required to rotate 65∘forward to the affected side

The FP radiographs were taken according to the introduction by Professor Lequesne. The patient was required to rotate 65∘forward to the affected side. (Fig. 1) On FP radiographs, the ACEA was measured by the angle between the line joining the midpoint of the femoral head to the anterior rim of the acetabular sourcil and the vertical line. (Fig. 4B) [39] The normal reference value for the ACEA is 20–40°. An ACEA less than 20° is defined as deficient anterior coverage, and an ACEA greater than 40° is defined as excessive anterior coverage [25].

**Fig. 2:**
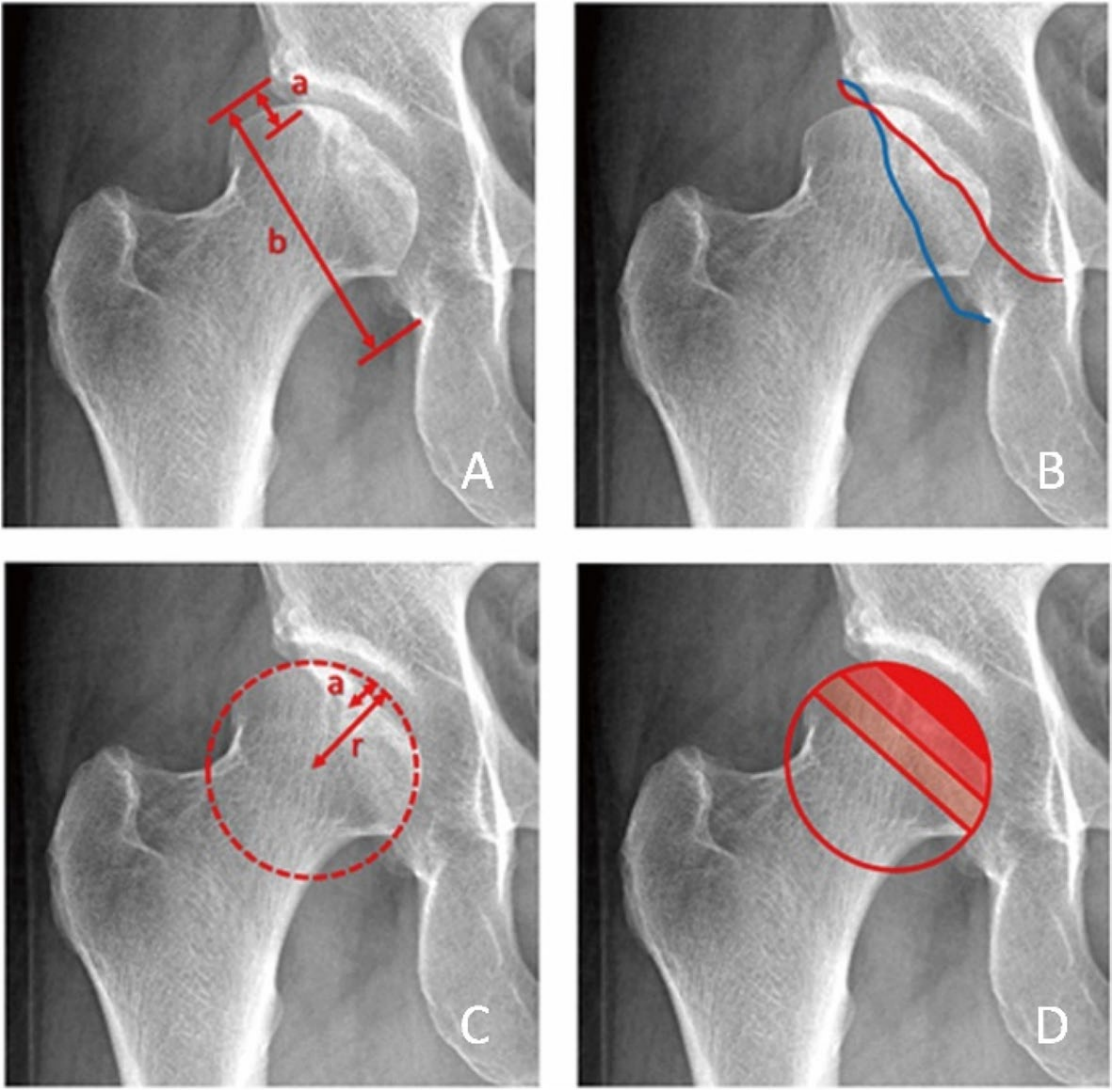
Four parameters for estimating the preacetabular anterior coverage were measured on standing pelvic AP radiographs. A. Crossover index: ratio of the width of the anterior acetabulum width (a) to the posterior wall width (b). B. Crossover sign: crossover between the anterior and posterior rims. C. AWI: The ratio of femoral head portion covered by anterior acetabulum (a) to femoral head radius (r). D. Rule of thirds: AWI 33%-66% is normal

The following parameters for estimating the preacetabular anterior coverage on standing pelvic AP radiographs were measured: the crossover index, the crossover sign [30], the anterior wall index [31], and rule of thirds [32] (Fig. 2).

The positive crossover sign is defined as crossover of the anterior and posterior rims of the acetabulum that can be identified on the AP radiograph. The crossover index is the ratio of the width of the anterior acetabulum (a) and the width of the posterior wall of the acetabulum (b) [23] (Fig. 2A). Because there is a high probability of a small crossover at the lateral acetabulum and at the anterior and posterior margins, in clinical practice, a crossover index with a value greater than 15% is generally defined as a positive crossover sign [30] (Fig. 2B).

The anterior wall index refers to the percentage of the area covered by the anterior rim of the acetabulum in the radius of the femoral head [40] (Fig. 2C). According to Professor Tannast [32, 41], the rule of thirds is utilized for categorizing the pelvic anterior coverage based on the width of the anterior acetabular wall covering the femoral head. The undercoverage is determined if the intersection point for the anterior wall is in the medial third of the femoral head radius, while excessive anterior coverage was determined if the intersection point is in the lateral third of the femoral head radius. Therefore, an AWI between 0 and 33% is defined as undercoverage, between 33 and 66% is defined as normal, and greater than 66% is defined as overcoverage (Fig. 2D).

All measurements were performed by two experienced hip surgeons who were able to perform periacetabular osteotomy independently.

To explore the relationship between the 4 parameters above and the ACEA in patients with hip dysplasia, we analyzed (1) the correlation between the crossover index and the ACEA, (2) the agreement of the classification of hips by the crossover sign and by the ACEA, (3) the correlation between the AWI and the ACEA, and (4) the agreement of the classification of hips by rule of thirds and by the ACEA. Bost preoperative and postoperative data were analyzed.

Statistical methods: SPSS 26 (IBM, Armonk, NK) statistical software was used for data analysis. The Kolmogorov–Smirnov (K-S) test determined the normal distribution of all of the measurement data. Pearson and Spearman correlation analysis was used to examine the correlation between the crossover index and the ACEA. Kendall's tau correlation analysis was conducted, and the result of the crossover sign was compared to the result of ACEA. The correlation between the anterior wall index and the ACEA was determined by Pearson correlation analysis. When hip joints were classified as undercovered, normal and overcovered by the rule of thirds, Kendall's tau correlation analysis was used to make comparisons between the classification and results of ACEA one by one. The alpha value was set to 0.05.

## Results

A total of 60 hips of 60 patients meet our inclusion criteria, while7 hips with deformities were excluded. Of the 7 excluded patients, 1 was due to multiple epiphyseal dysplasia (MED), 2 were due to severe coxa plana, 1 was due to severe hip joint deformation and subluxation caused by poliomyelitis, and 3 were due to severe hip subluxation due to cerebral palsy. None of our included hips had a treatment history. The study included 53 cases (53 hips) were included in the study, consisting of 6 males and 47 females, with an average age of 29.3 ± 8.6 (14–46) at the time of surgery.

The K-S test results showed that the preoperative and postoperative ACEA and AWI were normally distributed.

Preoperatively, according to the ACEA, while 41 hips had anterior undercoverage, while 12 hips had normal coverage, with an average ACEA was 9.0 ± 16.1° (-24.8° ~ 37.0°). Postoperatively, anterior undercoverage was detected in 3 hips, 40 hips had normal anterior coverage, and 10 hips were overcovered anteriorly with an average ACEA was 34.1 ± 9.3° (7.3°-60.4°).

Preoperatively, the average crossover index was 0.05 ± 0.10 (0.00 ~ 0.37), with no correlation with the ACEA (*P* = 0.66). Postoperatively, the mean crossover index was 0.03 ± 0.11 (0.00 ~ 0.57),with a correlation to the ACEA (*P* = 0.007). The correlation coefficient was 0.36.

Preoperatively, 9 hips had positive crossover signs, none of which had excessive anterior coverage according to the ACEA classification. Postoperatively, positive crossover signs were found in 4 hips, 3 of which had excessive anterior coverage according to the ACEA classification. Using the ACEA classification as the standard, the sensitivity was 0.30, and the specificity was 0.75. Postoperatively, a correlation was observed between the crossover sign and the ACEA after surgery (*P* = 0.003), and the correlation coefficient was 0.41.

**Fig. 3:**
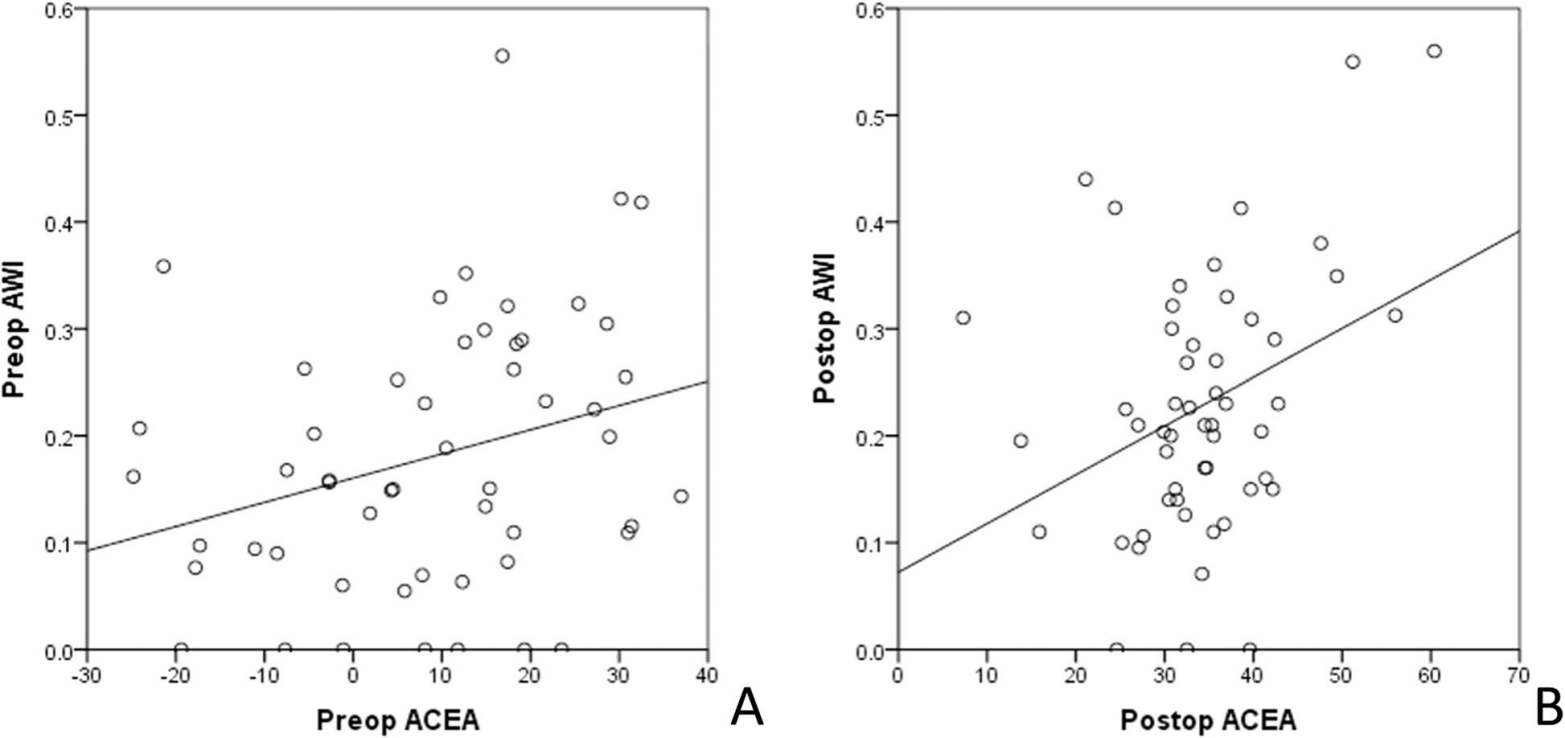
Correlation between the AWI and the ACEA. A. The AWI was correlated with the ACEA preoperatively (r = 0.288). B. The AWI was correlated with the ACEA postoperatively (r = 0.349)

As shown in Fig. 3, a preoperative AWI of 0.18 ± 0.13 (0.00 ~ 0.56) weakly correlated with the ACEA (P = 0.036, r = 0.288). The postoperative AWI was 0.23 ± 0.12 (0.00 ~ 0.56), which was also weakly correlated with the ACEA (*P* = 0.011, r = 0.349).

In this study, the average AWI of all of the patients with a normal ACEA (20–40°) was 0.21 ± 0.11 (0.00 ~ 0.44).

Preoperatively, 48 hips showed anterior undercoverage, while 5 hips had normal anterior coverage according to the classification of rule of thirds. Among the 53 hips, 41 hips agreed with the anterior coverage according to ACEA classifications (grey cells), and the accuracy was 77.36%. Nine hips (16.98%) were underestimated, and 3 hips (%) were overestimated. (Table 1) There was no correlation with the anterior coverage classified by the ACEA (P = 0.14). Postoperatively, deficient anterior coverage was detected in 42 hips, while 11 hips had normal anterior coverage according to the rule of thirds classification. No overcovered hip was detected. Among the 53 hips, only 10 hips agreed with the classification according to the ACEA (grey cells), with an accuracy of only 18.87%. The anterior coverage was underestimated in 43 hips (81.13%). (Table 2) There was no correlation between the anterior coverage classification by the rule of thirds and by the ACEA.

**Table 1:**
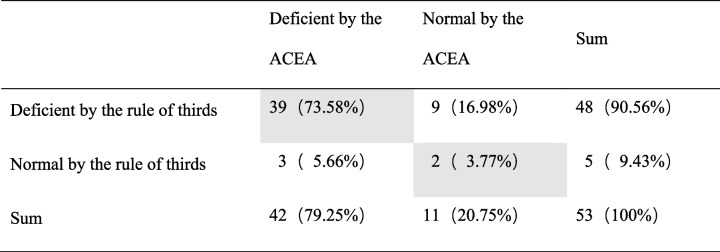
Comparison of the classification of hips by the rule of thirds and by the ACEA in preoperative patients with hip dysplasia

**Table 2:**
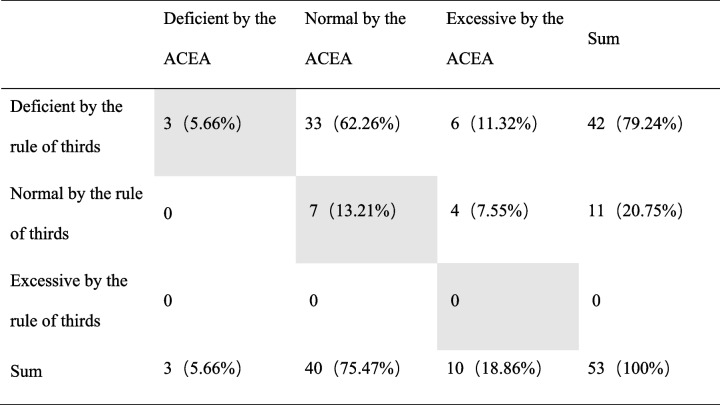
Comparison of the classification of hips by the rule of thirds and by the ACEA in postoperative patients with hip dysplasia

## Discussion

For hip dysplasia, the evaluation of the anterior coverage of the hip joint is as crucial as that of the lateral coverage. Currently, the ACEA measured on FP radiographs is widely used for evaluating anterior coverage. It has been proven to be related to clinical results [3, 9, 26–29]. However, radiologists are not always familiar with FP radiographs, and obtaining additional radiographs can be deemed redundant. Thus, hip surgeons often may prefer to use pelvic AP radiographs to estimate the anterior coverage instead.

The crossover index has been proven to be related to the ACEA in patients with Femoroacetabular impingement (FAI) [30]. However, to our knowledge, the correlation has not been studied in patients with hip dysplasia. In this study, the crossover index had no correlation with the ACEA preoperatively. However, after the deficiency was corrected in the surgery, there was a weak correlation. The postoperative result is similar to what has been described in patients with FAI.

In our study, using the crossover sign was used to detect anterior overcoverage in preoperative patients with hip dysplasia, none of the results agreed with the ACEA results. Postoperatively, some crossover sign results agreed with ACEA results, but there was an unsatisfactory specificity (75.0%) and a worse sensitivity (30.0%). Therefore, the positive crossover sign in the clinic might only be a reminder of excessive anterior coverage [42, 43] and should not be used as the decisive parameter.

The different methodologies used to detect excessive anterior coverage by ACEA and the crossover index/the crossover sign explain the low agreement between the two parameters. While a positive crossover sign means that the anterior acetabular wall is partially larger than the posterior acetabular wall, this can result from not only excessive coverage but also deficient posterior coverage.. Deficient posterior walls are frequently seen in patients with hip dysplasia, [44] which greatly affects the determination of the anterior coverage. Therefore, relying solely on the crossover sign to evaluate the anterior coverage is not recommended, as it may overestimate the anterior coverage. Additionally, the postoperative correlation between the crossover sign and the ACEA is due to the posterior wall, which decreases the error caused by the deficient posterior wall and increase the value of the crossover sign in determining anterior coverage increases.

**Fig. 4:**
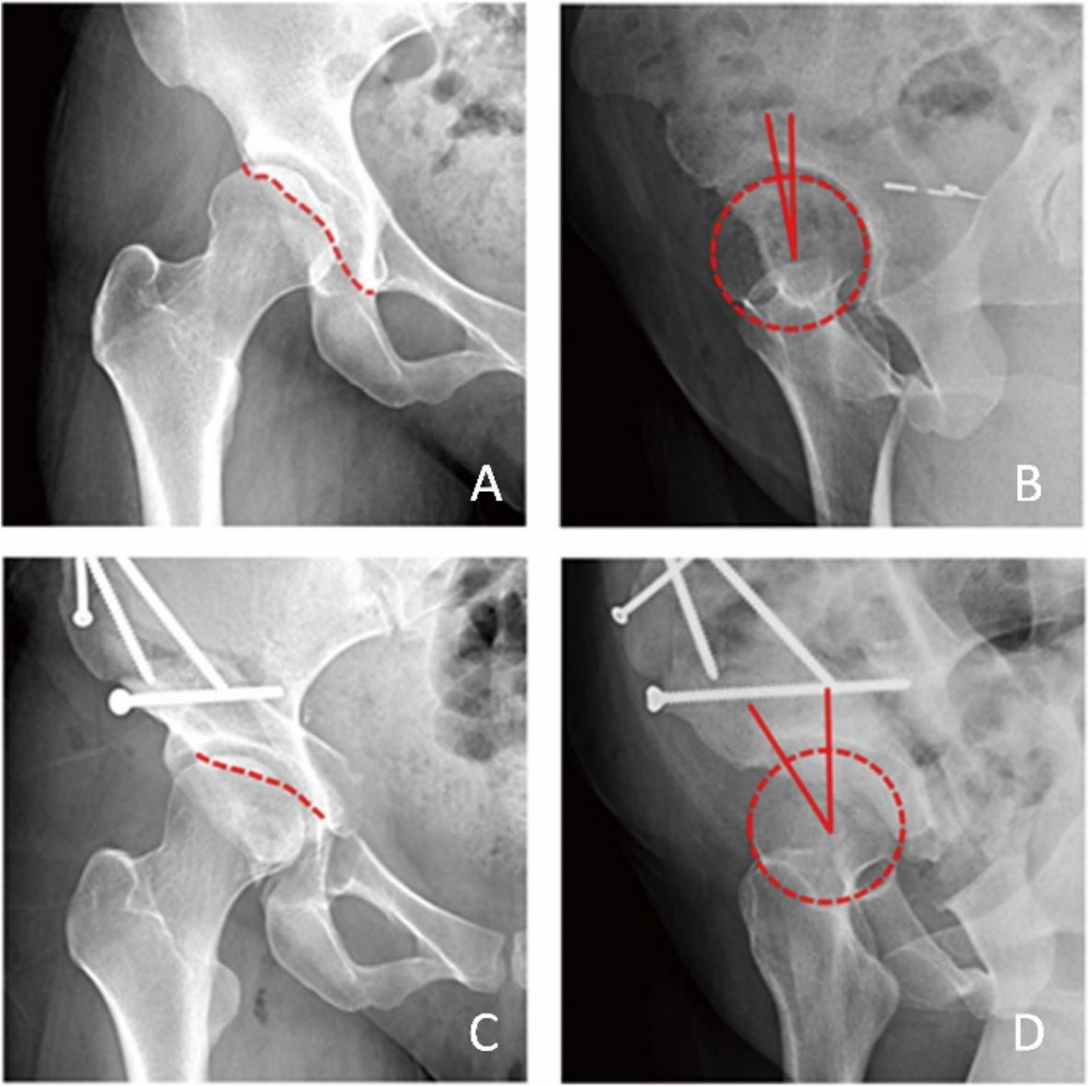
A patient with hip dysplasia. A. Preoperative pelvic AP radiograph of the anterior rim of the acetabulum with a normal appearance; the lateral rim of the AIIS was visible anterolateral to the anterior rim line of the acetabulum. B. Preoperative FP radiograph showing deficient anterior coverage of the acetabulum. C. Postoperative pelvic AP radiograph showing no obvious changes in the anterior rim of the acetabulum. D. Postoperative FP radiograph showing normal anterior coverage

When measuring the AWI to determine anterior coverage of the hip joint in patients with hip dysplasia, we used the location of the anterior rim of the acetabulum on the femoral head on the pelvic AP radiograph instead of the relationship between the anterior and posterior walls of the acetabulum used in the crossover sign and crossover index. The AWI measurement avoids the effect of a deficient posterior wall of the acetabulum. It is weakly but significantly correlated with the ACEA. When comparing the preoperative and postoperative data, it was found that the preoperative correlation was lower (Fig. 4). On the preoperative scatter plots of the AWI and ACEA, there are some cases showing a significant deviation from the regression line. When we traced these cases, some common characteristics were found. In all these cases, the lateral contours of the anterior acetabulum spines (AIIS) are clearly visible on pelvic AP radiographs, as demonstrated in Fig. 3A. On the FP radiograph, the anterior edge of the articular surface was located only slightly anterior to the top of the femoral head center, with insufficient supporting area (Fig. 4B). The prominent AIIS instead of the real anterior rim of the socket formed the seemingly normal anterior wall on the pelvic AP radiograph (Fig. 3A), leading to overestimation of the anterior coverage of the hip joint on the pelvic AP radiograph. This also explains why there is only a moderate correlation between AWI on pelvic radiographs and CT [45]. In the same patient, after the acetabulum was reoriented in PAO, the supporting area of the acetabulum rotated laterally and anteriorly, resulting in a significantly increase in the anterior hip coverage and the ACEA (Fig. 4D). However, the AWI did not lead to any changes on the pelvic AP radiographs (Fig. 4C).

For the above reasons, the weakness of estimating the anterior coverage on pelvic AP radiographs is that some of the anterior rims identified on AP radiographs are not the real anterior rims of the socket. The “Anterior walls” on AP radiographs are enlarged by the AIIS, which interferes with accurate measurement. This interference factor affected our determination of the anterior coverage evaluation to a certain extent.

**Fig. 5:**
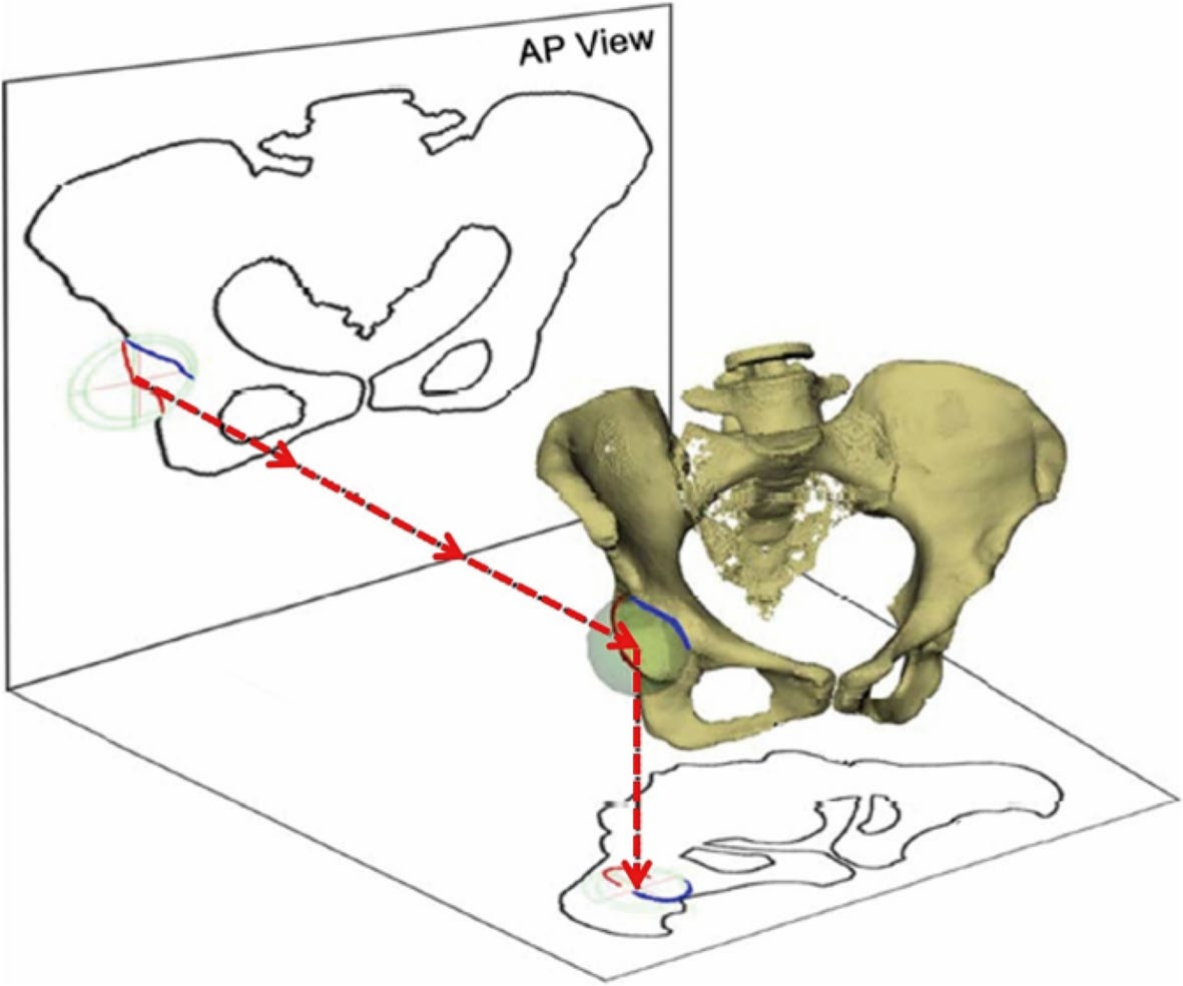
The working principle of Hip^2^Norm is to manually map the anterior and posterior rims of the acetabulum on a pelvic AP radiograph and then to calculate the anterior coverage of the hip joint on a statistical model

Furthermore, our study also found that the rule of thirds classification [46], which is also based on pelvic AP radiograph, has similar limitations as AWI. In this study, we did not replicate results from Professor Tannast. The rule of thirds was verified not by direct measurements of the ACEA or CT data but by the software Hip^2^Norm (University of Bern, Bern, Switzerland) [32, 41]. The Hip2Norm software calculates anterior acetabular coverage by manually tracing the anterior and posterior acetabular walls on a pelvic anterior–posterior (AP) radiograph. (Fig. 5). It then projects them onto a spherical acetabulum in a statistical model, and then onto the horizontal plane to determine anterior coverage. Thus, the accuracy of Hip2Norm's measurement of anterior coverage still relies on the accuracy of the identification of the anterior acetabular wall on the pelvic AP radiograph [47, 48]. Because Hip^2^Norm is also based on the AP radiograph, the mechanism is the same as AWI and the rule of thirds. Any factors that affect the determination of anterior coverage on pelvic AP radiographs will also affect the measurement results of Hip2Norm software. The underlying logic of all 3 methods is the same, so a perfect correlation between them does not mean that they reproduce anterior hip coverage perfectly.

**Fig. 6:**
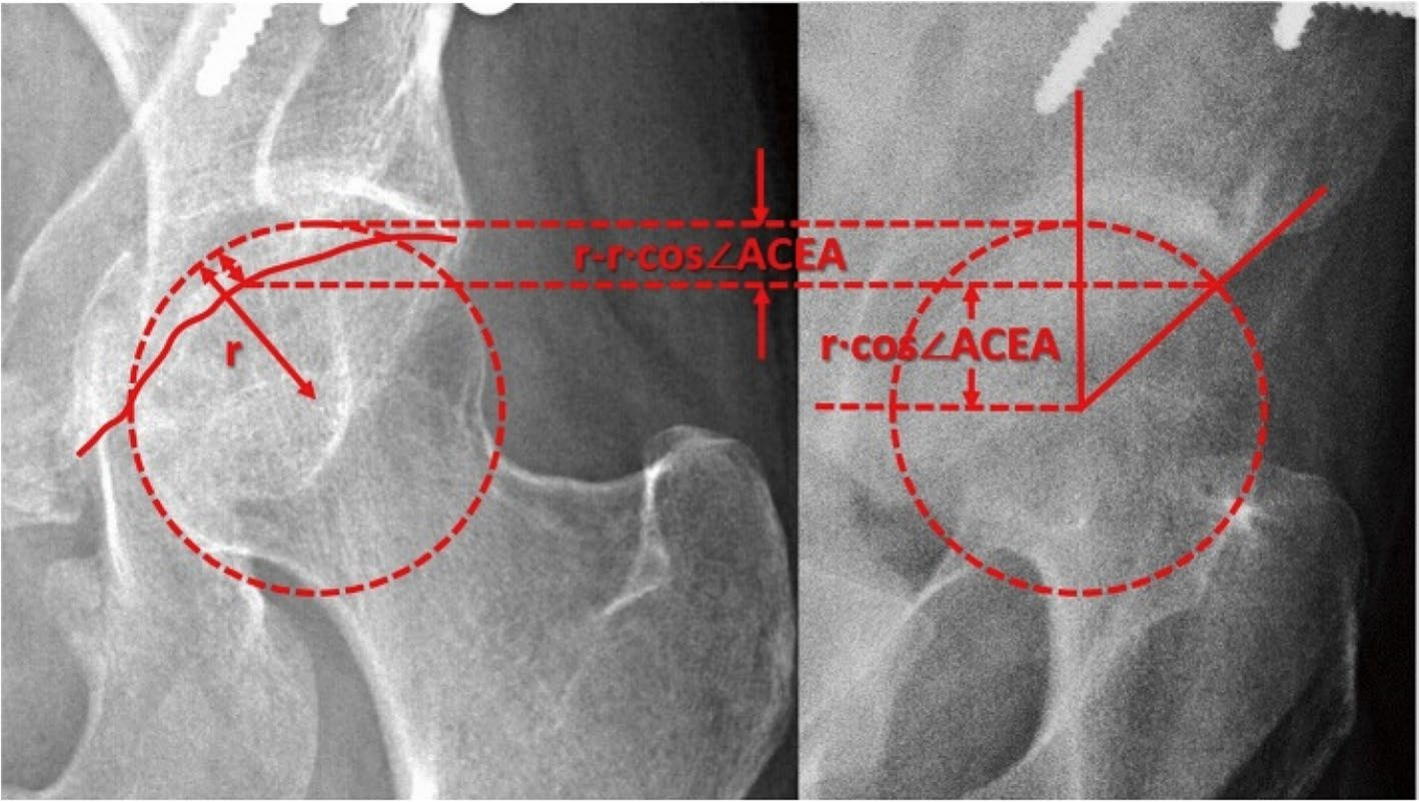
A rough conversion relationship of the AWI on a pelvic AP radiograph and the ACEA on an FP radiograph

The AIIS has a significant impact on the identification of the anterior rim of the joint surface on pelvic AP radiographs. Only when the AIIS is not prominent, the pelvic anterior wall that is visible on the radiograph coincides with the anterior wall of the socket.. Without the AIIS’ interference, we can see that the rule of thirds will slightly underestimate the anterior coverage with trigonometric calculation. The results of the calculations show that when the ACEA ranges from 20° to 40°, the AWI value ranges between 0.06 and 0.23 rather than ranging from 0.33–0.66, which would be recommended by the rule of thirds (Fig. 6). In this study, the AWI was 0.21 ± 0.11 (0.00 ~ 0.44) for all patients with an ACEA between 20 and 40°, which was also significantly lower than the reference value of 0.33–0.66. The anterior coverage of some hips was underestimated with 9 hips(16.98% of the total) being underestimated preoperatively and postoperative 34 hips (81.13%) postoperatively.

When the AIIS is prominent, the anterior rim identified on a pelvic AP radiograph is located laterally and inferiorly to the anterior border of the joint surface. Because it is impossible to visually distinguish the acetabular surface from the AIIS on the AP radiograph, measuring the anterior wall on a pelvic AP radiograph will result in overestimation of the anterior coverage. For these patients, there is a possibility of obtaining deficient postoperative anterior coverage if we correct the acetabulum based only on pelvic AP radiographs. If FP radiographs can be obtained before surgery, the correction of the anterior coverage can be more accurate (Fig. 3). For patients with hip dysplasia, a certain error rate is obtained when estimating the anterior coverage on pelvic AP radiographs.

Therefore, it is risky to evaluate anterior coverage using pelvic AP radiographs instead of FP radiographs.

There are some limitations in this study: 1. Because the accuracy of anterior coverage measured from CT data in the supine position was greatly affected by pelvic tilt [14, 18], the gold standard of anterior coverage should be anterior coverage measured on standing CT data. Because we do not perform hip CT on standing position right now, after careful consideration, we finally decided to use ACEA, a 2D parameter, as a standard of all the parameters. There are 2 reasons that we took this approach. (1) ACEA is taken in the patient’s standing position. It can reproduce the weight-bearing state of the hip joint in the standing position. (2) ACEA has been confirmed in clinical practice and widely accepted by most doctors in clinical practice [3, 9, 26–29]. The acetabulum is a three-dimensional structure, whereas the ACEA on an FP radiograph is only a two-dimensional parameter. We will try to solve this problem in subsequent studies.

## Conclusions

Determining the anterior coverage based on pelvic AP radiographs should be done with caution. It is recommended to take FP radiographs routinely for determining anterior hip coverage.

## Data Availability

Data available upon request. The contact should be made via the first author shenzhentie@163.com.

## Abbreviations

AP view: Pelvic anteroposterior view
FP view: False profile view
ACEA: Anterior center edge angle
AWI: Anterior wall index
PAO: Periacetabular osteotomy
FAI: Femoroacetabular impingement
K-S test: Kolmogorov–Smirnov test
MED: Multiple epiphyseal dysplasia
AIIS: Anterior acetabulum spines
